# Computing the partition dimension of certain families of Toeplitz graph

**DOI:** 10.3389/fncom.2022.959105

**Published:** 2022-10-14

**Authors:** Ricai Luo, Adnan Khalil, Ali Ahmad, Muhammad Azeem, Gafurjan Ibragimov, Muhammad Faisal Nadeem

**Affiliations:** ^1^School of Mathematics and Physics, Hechi University, Hechi, China; ^2^Department Computer Sciences, Al-Razi Institute Saeed Park, Lahore, Pakistan; ^3^College of Computer Science and Information Technology, Jazan University, Jazan, Saudi Arabia; ^4^Department of Mathematics, Riphah Institute of Computing and Applied Sciences, Riphah International University Lahore, Lahore, Pakistan; ^5^Department of Mathematics, Institute for Mathematical Research, Universiti Putra Malaysia, Serdang, Malaysia; ^6^Department of Mathematics, COMSATS University Islamabad, Lahore Campus, Lahore, Pakistan

**Keywords:** Toeplitz graph, resolving sets, constant partition dimension, bounds on partition dimension, partition resolving set

## Abstract

Let *G* = (*V*(*G*), *E*(*G*)) be a graph with no loops, numerous edges, and only one component, which is made up of the vertex set *V*(*G*) and the edge set *E*(*G*). The distance *d*(*u, v*) between two vertices *u, v* that belong to the vertex set of *H* is the shortest path between them. A *k*-ordered partition of vertices is defined as β = {β_1_, β_2_, …, β_*k*_}. If all distances *d*(*v*, β_*k*_) are finite for all vertices *v* ∈ *V*, then the *k*-tuple (*d*(*v*, β_1_), *d*(*v*, β_2_), …, *d*(*v*, β_*k*_)) represents vertex *v* in terms of β, and is represented by *r*(*v*|β). If every vertex has a different presentation, the *k*-partition β is a resolving partition. The partition dimension of G, indicated by *pd*(*G*), is the minimal *k* for which there is a resolving *k*-partition of *V*(*G*). The partition dimension of Toeplitz graphs formed by two and three generators is constant, as shown in the following paper. The resolving set allows obtaining a unique representation for computer structures. In particular, they are used in pharmaceutical research for discovering patterns common to a variety of drugs. The above definitions are based on the hypothesis of chemical graph theory and it is a customary depiction of chemical compounds in form of graph structures, where the node and edge represent the atom and bond types, respectively.

## 1. Introduction

Mathematics plays a key role in social science such as computer science, physics, and chemistry. If *L* = {*l*_1_, *l*_2_, …, *l*_*k*_} is a graph's ordered set of vertices and *v* ∈ *G*, then the *k*-tuple *r*(*v*|*L*) = (*r*(*v, l*_1_), *r*(*v, l*_2_), …, *r*(*v, l*_*k*_)). The notation *r* is the representation of *v* with regard to *L*, and the symbol *L* is said to be a resolving set if the different vertices of *G* have different representations regard to *L*. *H*'s metric dimension, indicated by *dim*(*H*), is the minimal number of vertices in the resolving set. The task of computing a graph's locating set is a Non-deterministic Polynomial time problem or NP-hard (Lewis et al., 1983). These ideas have been mentioned in the literature (Chvatal, 1983; Slater, 1988; Khuller et al., 1996; Chartrand et al., 2000a, 2003; Buczkowski et al., 2003; Caceres et al., 2007).

Another form of dimension is partition dimension, which is similar to the metric dimension (Chartrand et al., 2000b) as follows: The *k*-ordered partition is designed as β = {β_1_, …, β_*k*_} and *r*(*v*|β) = {*d*(*v*, β_1_), *d*(*v*, β_2_), …, *d*(*v*, β_*k*_)} are named as *k*-tuple representations. If each *v* in *V*(*G*) has a unique representation with regard to β, then the resolving partition of the vertex set is termed β, and the least value of the resolving partition set of *V*(*G*) is called the partition dimension of *G* and is indicated as *pd*(*G*) (Chartrand et al., 2000b). The metric dimension problem's computational complexity and NP-hardness were studied in Lewis et al. (1983). Because computing the *pd* is a more advanced variant of computing the metric dimension, it is likewise an NP-complete task. For simple graphs, there is a well-known inequality between *dim*, and *pd* (Chartrand et al., 2000b).


(1) $$\begin{array}{l} pd\left(G\right)\leq dim\left(G\right)+1. \end{array}$$

**Figure 1:**
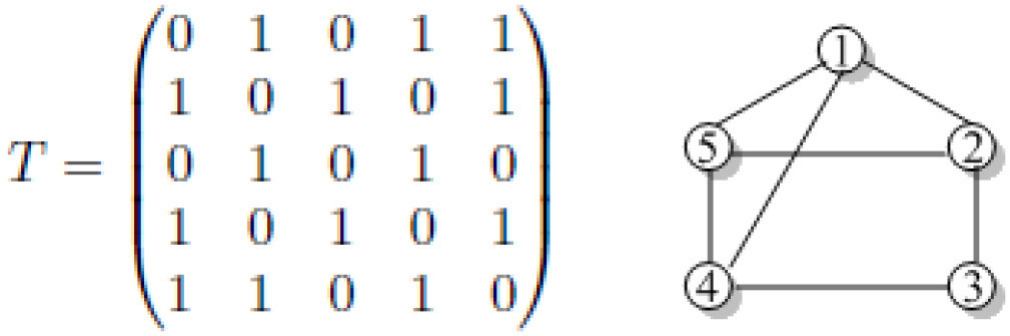
The corresponding Toeplitz graph and the adjacency matrix *T* *T*_5_〈1, 3, 4〉.

A *n* × *n* matrix *A* = *a*_*xy*_ is a Toeplitz matrix if *a*_*xy*_ = *a*_*x*+1,*y*+1_ for each *x, y* = 1, 2, ..., *n* − 1. A loopless and having no multi-edges graph termed as *T*_*n*_ is Toeplitz graph if the matrix is the symmetric Toeplitz matrix. The Topelitz graph *T*_*n*_〈*t*_1_, *t*_2_, *t*_3_, …, *t*_*p*_〉, where 0 < *t*_1_ < *t*_2_ < …*t*_*p*_ < *n* with *V*(*H*) = {1, 2, 3, …, *n*} has *E*(*H*) = {(*x, y*), 1 ≤ *x* ≤ *y* ≤ *n*}, *iff* *y* − 1 = *t*_*q*_ for some *q*, 1 ≤ *q* ≤ *p* (Liu et al., 2019). Let *n* = 5, *k* = 2, *t*_1_ = 1, *t*_2_ = 3, *and t*_3_ = 4. Figure 1 highlight the adjacency matrix *T* and its corresponding Toeplitz graphs *T*_5_〈1, 3, 4〉.

Toeplitz matrices play a major role in physical data-processing and in determining the discrete form of an integral and differential equations are considered as applications. Furthermore, matrices also contributed in process of stationary, the theories of polynomials of orthogonals and moment problem (Heinig and Rost, 1984) for more details reader can see (Ku and Kuo, 1992; Hua et al., 2010).

The researchers in Harary and Melter (1976) founded the concept of resolvability in graphs. Chartrand et al. (2000b) first time introduced the concept of pd. Javaid and Shokat (2008) discussed the pd of wheel graphs. Yero and Velázquez (2010) computed the pd of the cartesian product of graphs. Fehr et al. (2006) disproved a conjecture regarding the pd of products of graphs. The upper bound for the pd of the parallel composition of any graph was studied by Mohan et al. (2019). They also came up with an exact solution for the parallel composition of pathways of various lengths. Some updated references are (Ahmad et al., 2021; Ali et al., 2021; Azeem et al., 2021, 2022; Shanmukha et al., 2022a,b,c; Usha et al., 2022).

Resolvability of the graph has application in many fields of science such as in chemistry for representing chemical compounds (Browsable, 1998), Djokovic-Winkler relation (Caceres et al., 2007), strategies for the mastermind game (Chvatal, 1983), pattern recognition and image processing, hierarchical data structures (Melter and Tomescu, 1984), and robots navigation in networks (Khuller et al., 1994). For a better understanding of this topic, some very detailed articles are (Chartrand et al., 2000b; Saenpholphat and Zhang, 2002; Javaid et al., 2012; Velazquez et al., 2012; Velázquez et al., 2014; Yero et al., 2014; Siddiqui and Imran, 2015; Alatawi et al., 2022; Alshehri et al., 2022; Khabyah et al., 2022; Koam et al., 2022,a,b; Nadeem et al., 2022).

The theorems that follow are quite useful for calculating the *pd* of graphs.

**Theorem 1** (Chartrand et al., 2000b). “Let *G* be a connected graph of order *n* ≥ 2. Then *pd*(*G*) = 2 if and only if *G* = *P*_*n*_”.

**Theorem 2** Chartrand et al. (2000b) “Let *ϕ* be a resolving partition of ε(⋎) and ϵ_1_, ϵ_2_ ∈ ε(⋎). If *d*(ϵ_1_, *w*) = *d*(ϵ_2_, *w*) for all vertices *w* ∈ ε(⋎)\(ϵ_1_, ϵ_2_), then ϵ_1_, ϵ_2_ belong to different classes of *ϕ*.”

This study's findings the *pd* of Toeplitz graph with two generators 1 and *t* in Section 2 and Toeplitz graph partition dimension with three generators 1, 2, and *t* in Section 3.

## 2. Partition dimension of *T*_*n*_〈1, *t*〉

The coming section is containing the discussion on the *pd* of the Toeplitz graph *T*_*n*_〈1, *t*〉, for *t* ≥ 2 the *pd* of the graph is three.

**Theorem 2.1**. *A Toeplitz graph with* *n* ≥ 4 *is* *T*_*n*_〈1, 2〉. *After that*, *pd*(*T*_*n*_〈1, 2〉) = 3.

*Proof*. Let the Toeplitz graph with *n* ≥ 4 is *T*_*n*_〈1, 2〉 Then we will show that the Toeplitz graph with generators 1 and 2 consist a resolving partition set, β = {β_1_, β_2_, β_3_} with three elements, where β_1_ = {*v*_1_}, β_2_ = {_*v*_*k*_}*k*≡0(*mod* 2)_, β_3_ = {_*v*_*k*_}*k*≡1(*mod* 2)_. Let β = {β_1_, β_2_, β_3_} resolve the vertices of graph *G* with *V*(*G*) = β_1_ ∪ β_2_ ∪ β_3_.

When *k* = 1, 2, …, *n*. In terms of resolving partition set β, we have the following representations of *v*_*k*_.


$$r(v_{k}|\beta )=\left(\left\lfloor \frac{k}{2}\right\rfloor ,\frac{{\left(-1\right)}^{k+1}+1}{2},\{\begin{array}{ll} 1 & k=1\\ \frac{{\left(-1\right)}^{k}+1}{2} & k\geq 2 \end{array}\right)$$

Because all of the representations of different vertices are distinct


(2) $$\begin{array}{l} pd(T_{n}\text{〈}1,2\text{〉})\leq 3. \end{array}$$

Conversely: Now, we will show that *pd*(*T*_*n*_〈1, 2〉) ≥ 3. Suppose on contrary that *pd*(*T*_*n*_〈1, 2〉) = 2. We know that *pd*(*G*) = 2, iff *G* is a path graph by Theorem 1, it is not possible for *T*_*n*_〈1, 2〉. Thus,


(3) $$\begin{array}{l} pd\left(T_{n}\text{〈}1,2\text{〉}\right)\geq 3. \end{array}$$

Hence, from Inequalities (2) and (3), we have


$$\begin{array}{l} pd\left(T_{n}\text{〈}1,2\text{〉}\right)=3. \end{array}$$

**Theorem 2.2**. *Let a Toeplitz graph* *T*_*n*_〈1, 3〉 *with* *n* ≥ 5. *Then* *pd*(*T*_*n*_〈1, 3〉) = 3.

*Proof*. Let a Toeplitz graph *T*_*n*_〈1, 3〉 with *n* ≥ 5. We will show that the Toeplitz graph with generators 〈1, 3〉 consist of a resolving partition set, β = {β_1_, β_2_, β_3_} with three elements, where β_1_ = {*v*_1_}, β_2_ = {*v*_2_, …, *v*_*t*_}, β_3_ = {*v*_*t*+1_, …, *v*_*n*_}. There are two cases for β:

Case 1: If 1 ≤ *k* ≤ 3, then we can write the representation of *v*_*k*_ with respect to β as


$$r\left(v_{k}|\beta \right)=\left(k-1,q,1\right)$$

where $q=\left\lfloor \frac{1}{k}\right\rfloor ,$ this shows that all the representations are different so β resolves the vertex set of graph *T*_*n*_〈1, 3〉.

Case 2: If 4 ≤ *k* ≤ *n*, then we can write the representation of *v*_*k*_ with respect to β a


$$\begin{array}{l} r\left(v_{k}|\beta \right)=\left(q+j,q,0\right) \end{array}$$

where $q=\left\lceil \frac{k-3}{3}\right\rceil$ and *k* − 1 ≡ *j*(*mod* 3), this shows that all the representations are different, thus,


(4) $$\begin{array}{l} pd\left(T_{n}\text{〈}1,3\text{〉}\right)\leq 3. \end{array}$$

Conversely: We will prove that *pd*(*T*_*n*_〈1, 3〉) ≥ 3. On contrary suppose that *pd*(*T*_*n*_〈1, 3〉) = 2. Theorem 1 demonstrates that *pd*(*G*) = 2, iff *G* is a path graph, then it is not possible for *T*_*n*_〈1, 3〉. Thus,


(5) $$\begin{array}{l} pd\left(T_{n}\text{〈}1,3\text{〉}\right)\geq 3. \end{array}$$

Hence, from Inequalities (4) and (5), we have


$$\begin{array}{l} pd\left(T_{n}\text{〈}1,3\text{〉}\right)=3. \end{array}$$

**Theorem 2.3**. *Let a Toeplitz graph with notation* *T*_*n*_〈1, *t*〉 *with even generator* *t* ≥ 4, *n* ≥ *t*+2. *Then* *pd*(*T*_*n*_〈1, *t*〉) = 3.

*Proof*. Let a Toeplitz graph with notation *T*_*n*_〈1, *t*〉 with even generator *t* ≥ 4, *n* ≥ *t*+2. The Toeplitz graph with generators 〈1, *t*〉 consisting of a resolving partition set will be demonstrated. β = {β_1_, β_2_, β_3_}, where β_1_ = {*v*_1_}, $\beta_{2}=\left\{v_{\frac{t+2}{2}}\right\},$ β_3_ = {∀*v*_*k*_|*v*_*k*_ ∉ β_1_, β_2_}. There are three cases with respect to *v*_*k*_, which are the following;

Case 1: When $k\equiv 2,3,\ldots ,\frac{t}{2}\left(mod\;t\right).$ We have the following representation of *v*_*k*_ with regard to resolving partition set β;


$$\begin{array}{l} r\left(v_{k}|\beta \right)=\left(k-\rho t+\rho -1,\frac{\left(2\rho +1\right)t+2\left(\rho -k+1\right)}{2},0\right) \end{array}$$

where $\rho =\left\lfloor \frac{k}{t}\right\rfloor .$

Case 2: When $k\equiv \frac{t+2}{2}\left(modt\right).$ We have the following representation of *v*_*k*_ with respect to resolving partition set β;


$$\begin{array}{l} r\left(v_{k}|\beta \right)=\left(k-\rho t+\rho -1,\frac{2\left(\rho +k-1\right)-\left(2\rho +1\right)t}{2},z\right) \end{array}$$

where $\rho =\left\lfloor \frac{k}{t}\right\rfloor ,$ *z* = 1 when $k=\frac{t+2}{2}$ and otherwise *z* = 0.

Case 3: When $k\equiv 0,1,\frac{t+4}{2},\frac{t+6}{2},\ldots ,t-1\left(mod\;t\right).$ We have the following representation of *v*_*k*_ with respect to resolving partition set β;


$$\begin{array}{l} r\left(v_{k}|\beta \right)=\left(\rho t-k+\rho +1,\frac{2\left(\rho +k-2\right)-\left(2\rho -1\right)t}{2},z\right) \end{array}$$

where $\rho =\left\lfloor \frac{2k+t}{2t}\right\rfloor ,$ *z* = 1 when *k* = 1 and otherwise *z* = 0.

It is clear that no two vertices have the same representation, implying that there are not any two vertices with the same representation.


(6) $$\begin{array}{l} pd\left(T_{n}\text{〈}1,t\text{〉}\right)\leq 3. \end{array}$$

On contrary, we shall now demonstrate that *pd*(*T*_*n*_〈1, *t*〉) ≥ 3. Suppose on the contrary that *pd*(*T*_*n*_〈1, *t*〉) = 2. We know that by Theorem 1, it is not possible for even *t* of graph *T*_*n*_〈1, *t*〉. Thus,


(7) $$\begin{array}{l} pd\left(T_{n}\text{〈}1,t\text{〉}\right)\geq 3. \end{array}$$

Hence, from Inequalities (6) and (7), we have


$$\begin{array}{l} pd\left(T_{n}\text{〈}1,t\text{〉}\right)=3. \end{array}$$

**Theorem 2.4**. *Let a Toeplitz graph* *T*_*n*_〈1, *t*〉 *with odd* *t* ≥ 5, *n* ≥ *t*+2. *Then* *pd*(*T*_*n*_〈1, *t*〉) = 3.

*Proof*. Let a Toeplitz graph *T*_*n*_〈1, *t*〉 with odd *t* ≥ 5, *n* ≥ *t*+2. We will show that the Toeplitz graph with generators 1 and *t*, consists of a resolving partition set, β = {β_1_, β_2_, β_3_} with three elements, where β_1_ = {*v*_1_, *v*_2_, *v*_*t*+1_}, $\beta_{2}=\left\{v_{\frac{t+1}{2}},v_{\frac{t+3}{2}}\right\},$ β_3_ = {∀*v*_*k*_|*v*_*k*_ ∉ {β_1_, β_2_}}.

There are two cases for β_1_


$$\begin{array}{l} r(v_{k}|\beta_{1})\\ =\{\begin{array}{ll} k-2+q_{1}(1-t), & k\equiv 2,\ldots ,\frac{t+1}{2}+1(mod\;t)\\ t+1-k+(t+1)\left(q_{2}-s\right)+s, & k\equiv 0,1,\frac{t+1}{2}+2,...,t-1(mod\;t) \end{array} \end{array}$$

where $q_{1}=\left\lfloor \frac{k}{t}\right\rfloor ,$ $q_{2}=\left\lfloor \frac{k}{t+2}\right\rfloor$, and $s=\left\lfloor \frac{1}{k}\right\rfloor .$

There are two cases for β_2_


$$\begin{array}{l} r(v_{k}|\beta_{2})\\ =\{\begin{array}{ll} t+1-k+(t+1)q_{1}-s, & k\equiv 2,3,\ldots ,\frac{t+1}{2}(mod\;t)\\ \;k-1-\left(\frac{t+1}{2}\right)+q_{2}(1-t)+st, & k\equiv 0,1,\frac{t+1}{2}+1,\ldots ,t-1(mod\;t) \end{array} \end{array}$$

where $q_{1}=\left\lfloor \frac{k}{t}\right\rfloor$ and $s=\frac{t+1}{2}.$ where $q_{2}=\left\lfloor \frac{k}{t+2}\right\rfloor$ and $s=\left\lfloor \frac{1}{k}\right\rfloor .$

For β_3_, we have the following values


$$\begin{array}{ll} r(v_{k}|\beta_{2}) & =\{\begin{array}{ll} 2 & fork=1\\ 1 & fork=\frac{t+1}{2},\frac{t+3}{2},t+1\\ 0 & otherwise\; \end{array} \end{array}$$

From all these cases of β_1_, β_2_, and β_3_


$$\begin{array}{l} r\left(v_{k}|\beta \right)=\left(r\left(v_{k}|\beta_{1}\right),r\left(v_{k}|\beta_{2}\right),r\left(v_{k}|\beta_{3}\right)\right) \end{array}$$

We conclude that all representations are unique, and no two vertices have identical representations.


(8) $$\begin{array}{l} pd\left(T_{n}\text{〈}1,t\text{〉}\right)\leq 3 \end{array}$$

In contrary, we shall now demonstrate that *pd*(*T*_*n*_〈1, *t*〉) ≥ 3. Suppose on the contrary that *pd*(*T*_*n*_〈1, *t*〉) = 2. We know that by Theorem 1, it is not possible for odd *t* of graph *T*_*n*_〈1, *t*〉. So


(9) $$\begin{array}{l} pd\left(T_{n}\text{〈}1,t\text{〉}\right)\geq 3 \end{array}$$

Hence, from Inequalities (8) and (9), we can say that


$$\begin{array}{l} pd\left(T_{n}\text{〈}1,t\text{〉}\right)=3 \end{array}$$

## 3. Partition dimension of *T*_*n*_〈1, 2, *t*〉

In this section, we are going to discuss the partition dimension of *T*_*n*_〈1, 2, *t*〉. If *t* = 3, 4, 5, and *t* = 2*i*, *i* ≥ 3, *n* ≥ *t* + 2 then partition dimension is 4.

**Theorem 3.1**. *Let* *T*_*n*_〈1, 2, *t*〉 *be a Toeplitz graph*. *Then* *pd*(*T*_*n*_〈1, 2, *t*〉) = 4.

*Proof*. We split our theorem into three cases.

Case A: When *t* = 3, 4, 5.

Let *T*_*n*_〈1, 2, *t*〉 be a Toeplitz graph with *t* = 3, 4, 5, *n* ≥ *t*+2, then we will show that vertices of the Toeplitz graph with three generators consist of a resolving partition set, β = {β_1_, β_2_, β_3_, β_4_} where β_1_ = {*v*_1_}, β_2_ = {*v*_2_}, β_3_ = {*v*_3_, …, *v*_*t*_}, and β_4_ = {*v*_*t*+1_, …, *v*_*n*_}. Then there are the three cases that follow:

Case 1: If *k*≡1(*mod t*), then we can write the unique position of *v*_*k*_ regarding β as;


$$\begin{array}{l} r\left(v_{k}|\beta \right)=\left(q-k+1,q-k+2-\left\lfloor \frac{3}{t}\right\rfloor s,q-k+2-s,w\right) \end{array}$$

where $q=\left(t+1\right)\left\lfloor \frac{k}{t}\right\rfloor ,$ $s=\frac{k-1}{k}$, and $w=\left\lfloor \frac{t}{k+t-1}\right\rfloor .$ This shows that all the representations are different so β resolves the vertices of *T*_*n*_〈1, 2, *t*〉

Case 2: If *k*≡2, 3(*mod t*), we can write the representations of *v*_*k*_ regarding β as;


$$\begin{array}{l} r\left(v_{k}|\beta \right)=\left(q+1,k-2+\left(1-t\right)q,q+\left\lfloor \frac{2}{k}\right\rfloor ,s\right) \end{array}$$

where $q=\left\lfloor \frac{k-1}{t}\right\rfloor$ and $s=\left\lfloor \frac{t}{k+t-3}\right\rfloor .$ This indicates that all the representations are different so β resolves the vertices of *T*_*n*_〈1, 2, *t*〉.

Case 3: If *k* ≡ 4, 5(*mod t*), we can write the representations of *v*_*k*_ with respect to β as


$$\begin{array}{l} r\left(v_{k}|\beta \right)=\left(q+2,\left\lfloor \frac{k}{t}\right\rfloor +\left\lfloor \frac{t}{5}\right\rfloor ,q,\left\lfloor \frac{t}{k}\right\rfloor \right) \end{array}$$

where $q=\left\lfloor \frac{k-1}{t}\right\rfloor .$ This shows that all the representations are different so β resolve the vertices. From all three cases, we conclude that


(10) $$\begin{array}{l} pd\left(T_{n}\text{〈}1,2,t\text{〉}\right)\leq 4 \end{array}$$

Case B: When *t* = 6, 8.

Let β = {β_1_, β_2_, β_3_, β_4_} be a resolving partition set. Where β_1_ = {*v*_1_}, β_2_ = {*v*_2_, *v*_*t*_}, β_3_ = {*v*_3_, …, *v*_*t*−2_}, and β_4_ = {*v*_*t*−1_, *v*_*t*+1_, …, *v*_*n*_}. We have different cases on *v*_*k*_, which are following;

There are two cases for β_1_;


$$\begin{array}{l} r(v_{k}|\beta_{1})\\ =\{\begin{array}{ll} \left\lfloor \frac{k}{2}\right\rfloor -\left(\frac{t-2}{2}\right)\left\lfloor \frac{k}{t}\right\rfloor -z_{1}, & k\equiv 1,2,3,4,5(mod\;t)\\ \left(\frac{t-2}{2}+6-k\right)+\left(t+1\right)\left\lfloor \frac{k-1}{t}\right\rfloor +z, & k\equiv 0,6,7,\ldots ,t-1(mod\;t) \end{array} \end{array}$$

where *z*_1_ = 1 when *k* = *even*, *z* = 1 when *k* ≡ 0(*mod* 8) and otherwise both are 0.

There are three cases for β_2_;


$$\begin{array}{l} r(v_{k}|\beta_{2})\\ =\{\begin{array}{ll} \left\lfloor \frac{k}{2}\right\rfloor -1-\left(\frac{t-2}{2}\right)\left\lfloor \frac{k}{t}\right\rfloor +z_{1}, & k\equiv 2,3,4(mod\;t)\\ \left(\frac{t-4}{2}+6-k\right)-\left\lfloor \frac{k}{2}\right\rfloor +\left(\frac{t+2}{2}\right)\left\lfloor \frac{k}{t}\right\rfloor +z, & k\equiv 5,\ldots ,t-1(mod\;t)\\ \left\lfloor \frac{k-1}{t}\right\rfloor , & k\equiv 0,1(mod\;t) \end{array} \end{array}$$

where *z* = 0 when 5 ≤ *k* ≤ *t*−1 and otherwise *z* = 2, and *z*_1_ has defined in β_1_.

There are three cases for β_3_;


$$r(v_{k}|\beta_{3})=\{\begin{array}{ll} \left\lfloor \frac{k+1}{2}\right\rfloor -\left(\frac{t-2}{2}\right)\left\lfloor \frac{k}{t}\right\rfloor , & k\equiv 1,2(mod\;t)\\ \left\lfloor \frac{k}{t}\right\rfloor , & k\equiv 3,\ldots ,t-2(mod\;t)\\ \left\lfloor \frac{k+1}{t}\right\rfloor , & k\equiv 0,t-1(mod\;t) \end{array}$$

There is the only case for β_4_;


$$\begin{array}{ll} r(v_{k}|\beta_{4}) & =\{\begin{array}{ll} 1 & k=1,\ldots t-4\\ 0 & otherwise \end{array} \end{array}$$

It is clear that no two vertices have the same representation, implying that there are no two vertices with the same representation.


(11) $$\begin{array}{l} pd\left(T_{n}\text{〈}1,2,t\text{〉}\right)\leq 4. \end{array}$$

Case C: When *t* = 2*i*, *i* ≥ 5.

Let β = {β_1_, β_2_, β_3_, β_4_} be a resolving partition set. Where β_1_ = {*v*_2_}, β_2_ = {*v*_6_}, β_3_ = {*v*_*a*_}, and β_4_ = {∀*v*_*k*_|*v*_*k*_ ∉ β_1_, β_2_, β_3_}. where $a=2\left\lceil \frac{t+6}{4}\right\rceil .$

The following is a representation of all vertices *v*_*k*_ with regard to the resolving partition set β.

There are two cases for β_1_;


$$\begin{array}{l} r(v_{k}|\beta_{1})\\ =\{\begin{array}{ll} \left\lfloor \frac{k-1}{2}\right\rfloor -\left(\frac{t-2}{2}\right)\left\lfloor \frac{k}{2}\right\rfloor , & k\equiv 3,4,\ldots ,t-3(mod\;t)\\ 3-\left\lfloor \left|\frac{k-t+2}{2}\right|\right\rfloor +\left(\frac{t+2}{2}\right)\left\lfloor \frac{k}{t+3}\right\rfloor +\left\lfloor \frac{1}{k}\right\rfloor , & k\equiv 0,1,2,t-2,t-1(mod\;t) \end{array} \end{array}$$

There are four cases for β_2_;


$$\begin{array}{ll} r(v_{k}|\beta_{2}) & =\{\begin{array}{ll} \left\lfloor \frac{3}{k}\right\rfloor -\left(\frac{t-2}{2}\right)\left\lfloor \frac{k}{t+1}\right\rfloor +\left\lfloor \frac{k-\frac{t}{2}}{2}\right\rfloor , & k\equiv 0,1(mod\;t)\;t=10,12\\ \left\lfloor \frac{3}{k}\right\rfloor +\left\lfloor \frac{5\left(k-1\right)}{t}\right\rfloor -4\left\lfloor \frac{k}{t+1}\right\rfloor , & k\equiv 0,1(mod\;t),t\geq 14\\ 2-\left\lfloor \frac{k-2}{2}\right\rfloor +\left(\frac{t+2}{2}\right)\left\lfloor \frac{k}{t}\right\rfloor , & k\equiv 2,3,\ldots ,6(mod\;t)\\ \left\lfloor \frac{k-5}{2}\right\rfloor -\left(\frac{t-2}{2}\right)\left\lfloor \frac{k}{t}\right\rfloor , & k\equiv 7,8,\ldots ,t-1(mod\;t) \end{array} \end{array}$$

There are four cases for β_3_;


$$\begin{array}{ll} r(v_{k}|\beta_{3}) & =\{\begin{array}{ll} \left\lceil \frac{t}{4}\right\rceil , & k=1,2\\ \left\lceil \frac{t+2}{4}\right\rceil -k+3, & k=3,4\\ \left\lfloor \frac{k-2a}{2}\right\rfloor -\left(\frac{t-2}{2}\right)\left\lfloor \frac{k-5}{t}\right\rfloor +z_{1}, & k\equiv 0,\ldots ,4,2a,2a+1,\ldots ,\\ & t-1(mod\;t),k\geq 2a\;\\ \frac{t-6}{2}-\left\lfloor \frac{k-5}{2}\right\rfloor +\left(\frac{t+2}{2}\right)\left\lfloor \frac{k}{t}\right\rfloor -z_{2}, & k\equiv 5,\ldots ,t-3(mod\;t)\; \end{array} \end{array}$$

where *z*_1_ = 0 when *k* = *even*, otherwise 1 and *z*_2_ = 1 when *k* = *odd*, otherwise 0.

There is the only case for β_4_;


$$r(v_{k}|\beta_{4})=\{\begin{array}{ll} 1, & k=2,6,a\\ 0, & otherwise\; \end{array}$$

It is clear that no two vertices have the same representation, implying that there does not exist two vertices with the same representation.


(12) $$\begin{array}{l} pd\left(T_{n}\text{〈}1,2,t\text{〉}\right)\leq 4. \end{array}$$

Converse A, B, and C:

We will show that *pd*(*T*_*n*_〈1, 2, *t*〉) ≥ 4. On contrary, suppose that *pd*(*T*_*n*_〈1, 2, *t*〉) = 3.

Different cases on behalf of our assumption that *pd*(*T*_*n*_〈1, 2, *t*〉) is 3. If $\beta^{n}=\left\{\beta_{1},\beta_{2},\beta_{3}\right\},$ where β^*n*^ consists of sets of different resolving partition set that are following:

Case 1: β_1_ = {*v*_1_, *v*_2_}, β_2_ = {*v*_3_, *v*_4_}, $\beta_{3}={\left\{v_{i}\right\}}_{i=5}^{i=n},$ then we have the following different vertices with same representation; $r\left(v_{3}|\beta^{n}\right)=r\left(v_{4}|\beta^{n}\right)=\left(1,0,1\right).$
Case 2: β_1_ = {*v*_1_, *v*_3_}, β_2_ = {*v*_2_, *v*_4_}, $\beta_{3}={\left\{v_{i}\right\}}_{i=5}^{i=n},$ then we have the following different vertices with same representation; $r\left(v_{2}|\beta^{n}\right)=r\left(v_{4}|\beta^{n}\right)=\left(1,0,1\right).$
Case 3: β_1_ = {*v*_1_, *v*_2_, *v*_3_}, β_2_ = {*v*_4_}, $\beta_{3}={\left\{v_{i}\right\}}_{i=5}^{i=n},$ then we have the following different vertices with same representation; $r\left(v_{2}|\beta^{n}\right)=r\left(v_{3}|\beta^{n}\right)=\left(0,1,1\right).$
Case 4: β_1_ = {*v*_1_, *v*_2_}, β_2_ = {*v*_3_}, $\beta_{3}={\left\{v_{i}\right\}}_{i=4}^{i=n},$then we have the following different vertices with same representation; $r\left(v_{1}|\beta^{n}\right)=r\left(v_{2}|\beta^{n}\right)=\left(0,1,1\right).$
Case 5: β_1_ = {*v*_1_}, β_2_ = {*v*_2_, *v*_3_}, $\beta_{3}={\left\{v_{i}\right\}}_{i=4}^{i=n},$ then we have the following different vertices with same representation; $r\left(v_{t+2}|\beta^{n}\right)=r\left(v_{t+3}|\beta^{n}\right)=\left(2,1,0\right).$
Case 6: β_1_ = {*v*_1_, *v*_2_, *v*_4_}, β_2_ = {*v*_3_}, $\beta_{3}={\left\{v_{i}\right\}}_{i=5}^{i=n},$ then we have the following different vertices with same representation; $r\left(v_{2}|\beta^{n}\right)=r\left(v_{4}|\beta^{n}\right)=\left(0,1,1\right).$
Case 7: β_1_ = {*v*_1_, *v*_3_, *v*_4_}, β_2_ = {*v*_2_}, $\beta_{3}={\left\{v_{i}\right\}}_{i=5}^{i=n},$ then we have the following different vertices with same representation; $r\left(v_{t+3}|\beta^{n}\right)=r\left(v_{t+3}|\beta^{n}\right)=\left(1,2,0\right).$
Case 8: β_1_ = {*v*_1_}, β_2_ = {*v*_2_, *v*_4_}, $\beta_{3}={\left\{v_{3},v_{i}\right\}}_{i=5}^{i=n},$ then we have the following different vertices with same representation; $r\left(v_{3}|\beta^{n}\right)=r\left(v_{6}|\beta^{n}\right)=\left(1,1,0\right).$
Case 9: β_1_ = {*v*_1_}, β_2_ = {*v*_2_, *v*_3_, *v*_4_}, $\beta_{3}={\left\{v_{i}\right\}}_{i=5}^{i=n},$ then we have the following different vertices with same representation; $r\left(v_{2}|\beta^{n}\right)=r\left(v_{3}|\beta^{n}\right)=\left(1,0,1\right).$
Case 10: β_1_ = {*v*_1_}, β_2_ = {*v*_2_, *v*_3_, *v*_5_}, $\beta_{3}={\left\{v_{3},v_{i}\right\}}_{i=5}^{i=n},$ then we have the following different vertices with same representation; $r\left(v_{t+3}|\beta^{n}\right)=r\left(v_{2t+1}|\beta^{n}\right)=\left(2,\left\lfloor \frac{t+1}{3}\right\rfloor ,0\right).$
Case 11: β_1_ = {*v*_1_, *v*_5_}, β_2_ = {*v*_2_, *v*_4_}, $\beta_{3}={\left\{v_{3},v_{i}\right\}}_{i=6}^{i=n},$ then we have the following different vertices with same representation; $r\left(v_{1}|\beta^{n}\right)=r\left(v_{5}|\beta^{n}\right)=\left(0,1,1\right).$
Case 12: β_1_ = {*v*_1_, *v*_4_}, β_2_ = {*v*_3_, *v*_5_}, $\beta_{3}={\left\{v_{2},v_{i}\right\}}_{i=6}^{i=n},$ then we have the following different vertices with same representation; $r\left(v_{1}|\beta^{n}\right)=r\left(v_{4}|\beta^{n}\right)=\left(0,1,1\right).$
Case 13: β_1_ = {*v*_1_, *v*_4_}, β_2_ = {*v*_2_, *v*_5_}, $\beta_{3}={\left\{v_{3},v_{i}\right\}}_{i=6}^{i=n},$ then we have the following different vertices with same representation; $r\left(v_{2}|\beta^{n}\right)=r\left(v_{4}|\beta^{n}\right)=\left(1,0,1\right).$
Case 14: β_1_ ≡ 1(*mod* 3), β_2_ ≡ 2(*mod* 3), β_3_ ≡ 0(*mod* 3), then we have the following different vertices with same representation; $r\left(v_{1}|\beta^{n}\right)=r\left(v_{4}|\beta^{n}\right)=r\left(v_{7}|\beta^{n}\right)=\left(0,1,1\right).$

According to the above cases, we can easily conclude that our supposition is wrong, and we can not resolve the vertices of *T*_*n*_〈1, 2, *t*〉 into three resolving partition sets. Thus,


(13) $$\begin{array}{l} pd\left(T_{n}\text{〈}1,2,t\text{〉}\right)\geq 4 \end{array}$$

Hence, from Inequalities (1-3) and (13), we can say that


$$\begin{array}{l} pd\left(T_{n}\text{〈}1,2,t\text{〉}\right)=4 \end{array}$$

## 4. Conclusion and open problems

In this study, we looked at different families of Toeplitz graphs and established that the partition dimension of each family is the constant, if the Toeplitz graph consists of two generators, then *pd*(*T*_*n*_〈1, *t*〉)=3, where *t* ≥ 2 and if Toeplitz graph consists of three generators, *pd*(*T*_*n*_(〈1, 2, *t*〉)) = 4, where *t* = 3, 5 and *t* = 2*i* and *i* ≥ 2.

**Figure 2:**
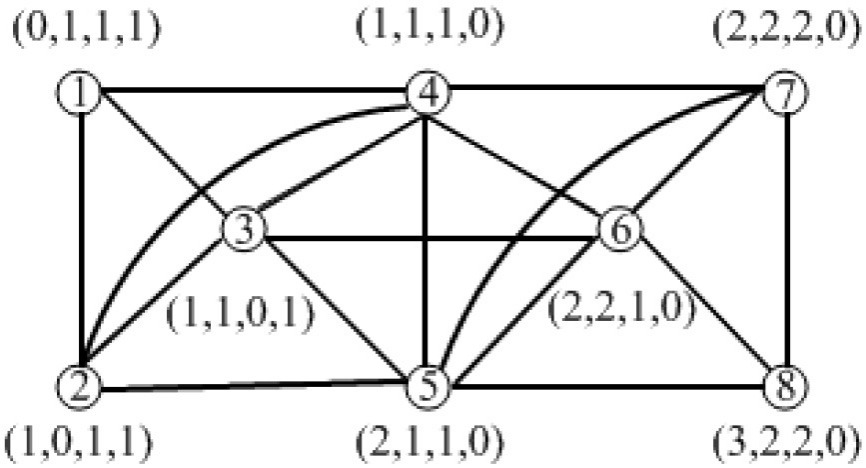
Toeplitz graph *T*_8_〈1, 2, 3〉 with partition dimension 4.

In this paper, inequality (1) also satisfied the metric dimension results (Liu et al., 2019) with our results for partition dimension (Figure 2).

**Open Problem 1.** *The partition dimension of the Toeplitz graph with two generators *k* ≥ 2, *s* ≥ 3 and *gcd*(*k, s*) = 1, is constant, bounded or unbounded?*

**Open Problem 2.** *The partition dimension of the Toeplitz graph with three generators *k* ≥ 2, *s* ≥ 3, *t* ≥ 4 and *gcd*(*k, s, t*) = 1, is constant, bounded or unbounded?*

**Open Problem 3.** *If the generators of the Toeplitz graph are increasing then the partition dimension either increasing or decreasing ?*

## Data availability statement

The original contributions presented in the study are included in the article/supplementary material, further inquiries can be directed to the corresponding authors.

## Author contributions

RL conceived of the presented idea. AK developed the theory and performed the computations. AA and MA verified the analytical methods. GI and MN investigated and supervised the findings of this work. All authors discussed the results and contributed to the final manuscript.

## Funding

This study was supported by the National Science Foundation of China (11961021 and 11561019), Guangxi Natural Science Foundation (2020GXNSFAA159084), and Hechi University Research Fund for Advanced Talents (2019GCC005).

## Conflict of interest

The authors declare that the research was conducted in the absence of any commercial or financial relationships that could be construed as a potential conflict of interest.

## Publisher's note

All claims expressed in this article are solely those of the authors and do not necessarily represent those of their affiliated organizations, or those of the publisher, the editors and the reviewers. Any product that may be evaluated in this article, or claim that may be made by its manufacturer, is not guaranteed or endorsed by the publisher.
